# One‐Step Fabrication of Non‐Ablative Microstructures for Efficient Second Harmonic Generation on LiNbO_3_ Thin Film

**DOI:** 10.1002/nap2.70096

**Published:** 2026-04-30

**Authors:** Feifei Wang, Yuntao Lang, Changji Pan, Yang Lu, Xiaozhe Chen, Rongke Gao, Zhe Peng, Liandong Yu

**Affiliations:** ^1^ College of Control Science and Engineering China University of Petroleum (East China) Qingdao China; ^2^ School of Mechanical Engineering and Automation Fuzhou University Fuzhou China; ^3^ Department of Physics ETH Zurich Zurich Switzerland

**Keywords:** femtosecond laser, metasurfaces, second harmonic generation

## Abstract

Lithium niobate (LiNbO_3_) is a key material for nonlinear optics applications, such as second harmonic generation (SHG), due to its strong nonlinear coefficient and broad transparency window. However, its high damage threshold makes it difficult to fabricate high‐quality microstructures, often resulting in low quality factors and poor SHG efficiency. Femtosecond lasers are well suited for processing transparent materials, but conventional ablation typically introduces surface cracks and scattering centers that degrade optical performance. In this study, we develop a one‐step femtosecond laser modification method to create periodic metasurfaces on LiNbO_3_ thin films without ablation. Compared to ablated structures, the laser‐modified regions exhibit minimal surface damage and avoid microcracking. The designed periodic structures facilitate efficient second‐harmonic generation through complementary mechanisms: the laser‐modified regions introduce a longitudinal focusing effect that locally enhances the optical field, whereas the periodic index modulation enables grating coupling to guided‐mode resonances under oblique incidence. Experimental results demonstrate a 4.75‐fold enhancement in SHG efficiency under 1030 nm femtosecond laser irradiation at oblique incidence. The mechanisms are analyzed using finite‐difference time‐domain simulation and Raman spectroscopy characterization. This work provides a simple and damage‐free strategy for manufacturing high‐performance nonlinear photonic devices, facilitating applications in integrated photonic circuits.

## Graphical abstract

This study presents a novel one‐step femtosecond laser modification technique for fabricating high‐quality periodic metasurfaces on lithium niobate thin films without surface damage and scattering centers. Under oblique incidence at 1030 nm, a 4.75‐fold enhancement in second‐harmonic generation efficiency is achieved through complementary mechanisms, including guided‐mode resonance and a longitudinal focusing effect within the laser‐modified zones.

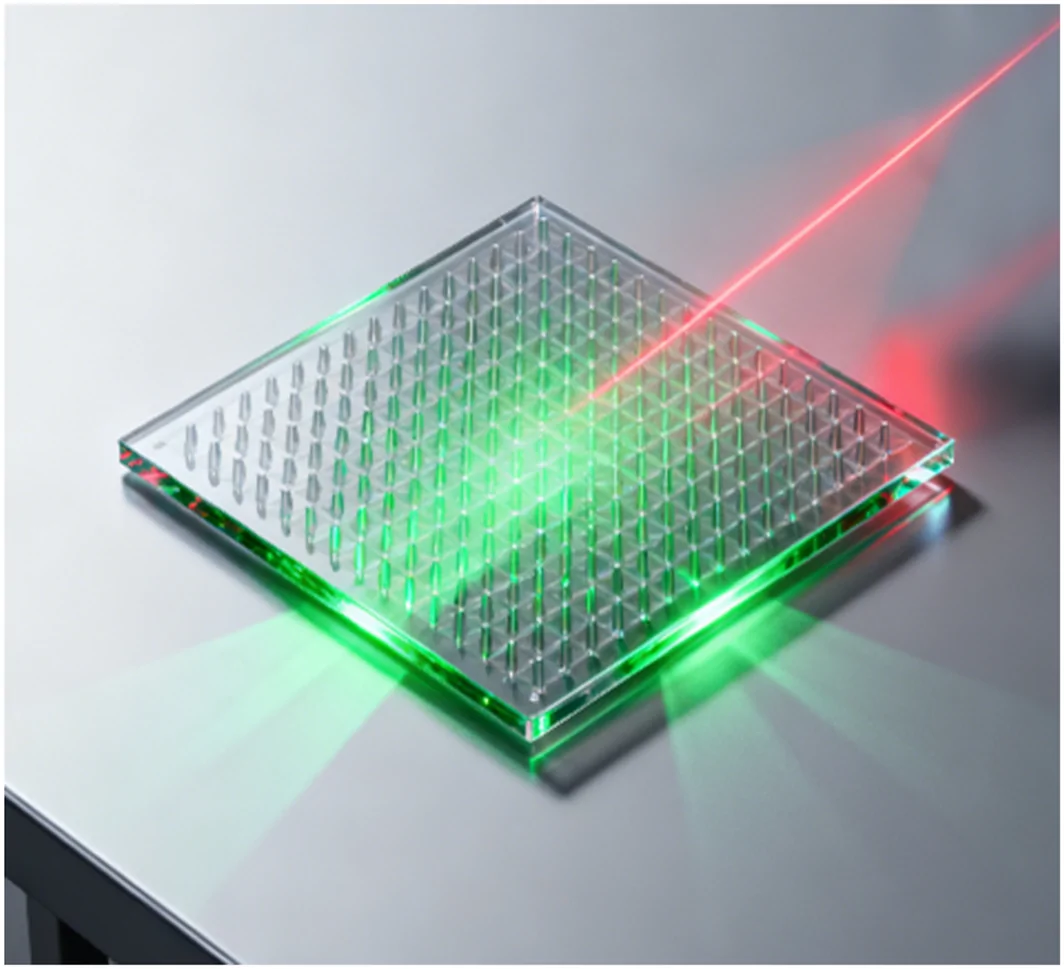

## 1 Introduction

The light‐materials interaction involves more than describing the linear relationship between induced polarization and the incident field, it necessitates consideration of the nonlinear effects of materials, which have wide applications in related fields such as materials science [1], optical imaging [2], and laser technology [3]. Frequency conversion is a key issue in nonlinear effects and can be used to generate coherent light for lasers that have no alternative wavelength. Generally, nonlinear birefringent crystals are used for responding to the nonlinear optical processes with sufficiently high optical intensities [4], such as second‐harmonic generation (SHG) [5], sum‐frequency generation, and difference‐frequency generation [6]. However, the nonlinear optical response of crystals is usually weak and only occurs in noncentrosymmetric crystals, which lack inverted center of symmetry in their cells [7]. Thus, strict phase‐matching conditions or quasi‐phase‐matching (QPM) conditions [8, 9, 10, 11], as well as sufficient light–matter interaction lengths, are required to facilitate nonlinear light generation. Over the years, it has been recognized that achieving proper phase matching is essential for maximizing the SHG efficiency, which involves using crystals with appropriate dimensions and nonlinear properties relative to the incident laser wavelength. Consequently, nonlinear devices tend to be bulky, which is contrary to the trend toward highly integrated on‐chip photonics technology. On the other hand, the phase‐matching condition can usually be ignored in nanoscale materials where the structure size is much smaller than the coherence length [12]. Therefore, finding more efficient nonlinear materials, such as QPM materials [13] or exploring mechanisms to enhance the nonlinear effects of materials [14, 15], has become a crucial focus in contemporary optical research.

This size‐dependent relaxation of phase‐matching requirements arises from the fact that in nanoscale materials where the structure size is much smaller than the coherence length, the phase‐matching condition can usually be ignored [12]. Even without strict phase matching, strong light–matter interactions can still enhance nonlinear efficiency. Recent studies have shown that all‐dielectric metasurfaces achieve this by leveraging resonant effects—such as guided‐mode resonances and Mie resonances—to strongly confine the optical field within subwavelength volumes, thereby increasing the local field intensity and boosting the efficiency of nonlinear processes such as second‐harmonic generation [16, 17, 18]. In metal‐based (plasmonic) metasurfaces, second‐harmonic generation primarily arises from plasma oscillations on the metal surface, which enable light compression into subwavelength volumes and can enhance nonlinear harmonic generation [19]. However, ohmic losses and the limited penetration of the magnetic field into the metal can substantially restrict conversion efficiency. To address these limitations, all‐dielectric metasurfaces have been proposed. These structures leverage high‐quality‐factor Mie resonances to confine light within low‐loss dielectric volumes, thereby amplifying the local field and enhancing nonlinear effects without suffering from metallic losses [20].

Lithium niobate (LiNbO_3_), known for its high nonlinear coefficient and magnetization [21], is transparent across a broad spectral range from ultraviolet to mid‐infrared wavelength. Its lack of central symmetry in the crystal structure imparts a significant second‐order nonlinear susceptibility, enabling efficient second‐harmonic generation. Recent advances in micro and nanofabrication techniques have provided new opportunities for exploring LiNbO_3_ in nonlinear nanophotonics [22, 23, 24, 25, 26]. Various resonant mechanisms have been exploited to enhance nonlinear optical responses in LiNbO_3_ metasurfaces. For instance, toroidal dipole resonance has been demonstrated in shallow‐etched LiNbO_3_ metasurfaces, where strong local field confinement leads to a ten‐fold enhancement of second‐harmonic generation compared to unpatterned lithium niobate on insulator [23]. Beyond simple resonance enhancement, nonlinear wavefront manipulation has also been achieved through a nonlinear metalens fabricated by a multiatmosphere cooperative etching technique, which focuses second harmonic waves at 390 nm to a sub‐micron spot with a 40‐fold peak intensity enhancement [24]. More recently, scalable fabrication of polycrystalline lithium niobate nanostructures with aspect ratios up to 6 has been realized using nanoimprint lithography, enabling the first lithium niobate nonlinear metalens with enhanced nonlinear signal intensity [25]. In parallel with experimental advances, theoretical proposals, such as etch‐free Si‐LN hybrid metasurfaces leveraging quasi‐bound states in the continuum, have predicted high‐efficiency second‐harmonic circular dichroism with conversion efficiency approaching ∼2 × 10^−3^ at moderate pump intensities [26]. Although existing nanofabrication techniques, such as inductively coupled plasma reactive ion etching [27] and focused ion beam milling [28], have successfully demonstrated submicron patterning of LiNbO_3_, these methods often involve multiple processing steps and can introduce surface damage. In contrast, femtosecond laser processing offers a simpler, one‐step alternative that enables rapid and cost‐effective fabrication of subwavelength structures with minimal surface damage, making it particularly attractive for rapid prototyping and scalable device fabrication [29]. Femtosecond lasers, characterized by ultrashort pulse durations and extreme peak intensities, are particularly suitable for processing transparent materials such as LiNbO_3_ [30]. Compared to ablative processing, which often produces cracks, debris, and noticeable surface topography, femtosecond laser modification results in less surface damage and reduced microcracking, which helps minimize scattering losses—an important consideration for nonlinear optical applications where field confinement is critical.

In this work, a one‐step femtosecond laser processing technique is employed to fabricate periodic subwavelength structures on LiNbO_3_ thin films via nonablative modification. Compared with conventional ablation‐based methods, this approach induces minimal surface damage and effectively suppresses microcrack formation, thereby maintaining the optical quality of the material. The designed periodic structures facilitate efficient second‐harmonic generation through complementary mechanisms: the laser‐modified regions introduce a longitudinal focusing effect that locally enhances the optical field under normal incidence, whereas under oblique incidence, the periodic index modulation enables grating coupling to guided‐mode resonances, resulting in strong in‐plane field confinement. Experimental results demonstrate a 4.75‐fold enhancement in SHG efficiency under 1030 nm femtosecond laser irradiation at oblique incidence. The physical mechanisms underlying the modification and enhancement are systematically investigated using finite‐difference time‐domain (FDTD) simulations and Raman spectroscopy. This strategy offers a straightforward and low‐damage route for developing high‐performance nonlinear photonic devices, with promising potential for applications in integrated photonic circuits and on‐chip wavelength conversion.

## 2 Experimental System

The experimental setup based on femtosecond laser precision processing periodic metasurface is illustrated in Figure 1a. High‐power femtosecond laser (PHAROS, Light Conversion Ltd.) with a central wavelength of 1030 nm and a pulse duration of 130 fs is used to irradiate LiNbO_3_ film for metasurface structuring and modification. LiNbO_3_ thin film samples (x‐cut) are sized 1 cm × 1 cm × 1 mm, consisting of a 500 nm‐thick LiNbO_3_ film, a 2 μm‐thick SiO_2_ buffer layer, and a Si substrate. The femtosecond laser pulses are focused vertically onto the sample surface along the *z*‐direction of the LiNbO_3_ film using a 50X microscope objective (NA = 0.65, Olympus Inc.). Laser fluence is adjusted using a neutral density attenuator. A high‐precision translation stage moves along the *x*‐direction at 1 Hz with a 1 μm periodicity to achieve single‐pulse processing of the sample.

**FIGURE 1:**
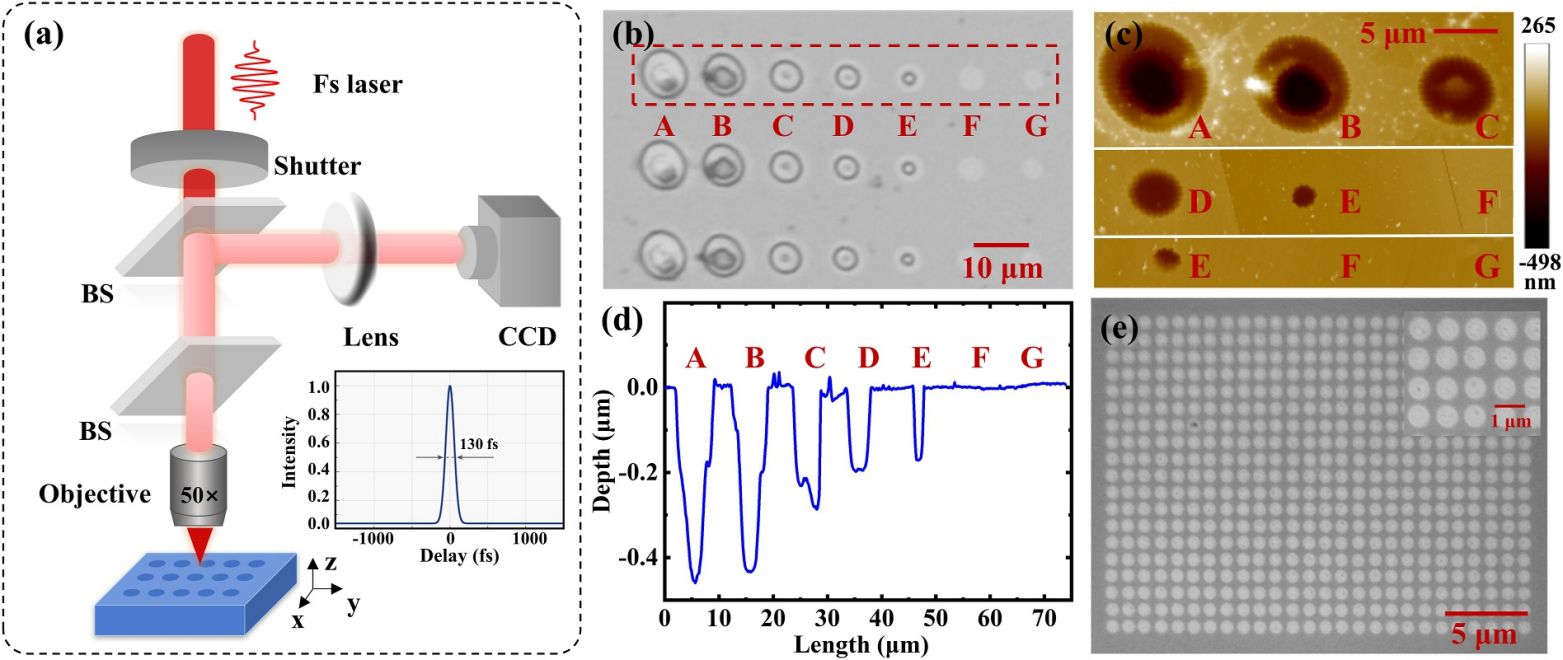
(a) Schematic of experimental setup for femtosecond laser processing; (b) optical microscopy images of laser‐processed and modified structures under single‐pulse energy from 1.2 to 0.5 μJ; (c) planar images of structures irradiated with various energies measured by AFM; (d) cross‐sectional views of the three‐dimensional structural dimensions; and (e) periodic metasurface modified structure diagram, with an inset showing magnified structure.

## 3 Results and Discussion

Figure 1b–e illustrate the surface structures of LiNbO_3_ films processed by femtosecond laser pulses at different fluences, which are measured by optical microscopy and atomic force microscope (AFM). The femtosecond laser single‐pulse energy is controlled from 1.2 to 0.5 μJ, resulting in structures labeled A‐G under optical microscopy as shown in Figure 1b. Figure 1c–d present the AFM images and cross‐sectional depth maps of the structures processed at various pulse energies. It can be observed that as the single‐pulse energy decreases to 0.5 μJ, the processed structures transition from deep ablation craters (480 nm deep) to a smoother state as seen in the F and G regions of Figure 1d. Under optical microscopy, as shown in Figure 1b, F and G regions manifest as modified regions, indicating that LiNbO_3_ film surface modification can be achieved by controlling the single‐pulse output energy. Figure 1e displays a periodic metasurface of the LiNbO_3_ film prepared by 0.5 μJ pulse energy, with a period of 1 μm and a diameter of 0.7 μm. To systematically investigate the enhancement of SHG conversion efficiency, we fabricated and characterized a series of structures with varying periods and feature sizes, the optical images are shown in Supporting Information S1: Figure S1.

To investigate the origin of the refractive index modification induced by femtosecond laser writing, Raman spectroscopy was performed on both the laser‐processed tracks and the surrounding unmodified regions using a confocal Raman microscope (InVia, Renishaw Inc.) with a 532 nm continuous‐wave laser (10 mW) focused through a 40X objective (N.A. = 0.75). As shown in Figure 2a, the Raman spectra collected from the irradiated zone (blue curve) and the pristine substrate (red curve) exhibit similar features, with only minor intensity reduction observed in the modified region, suggesting that the crystalline structure remains largely intact after laser processing.

**FIGURE 2:**
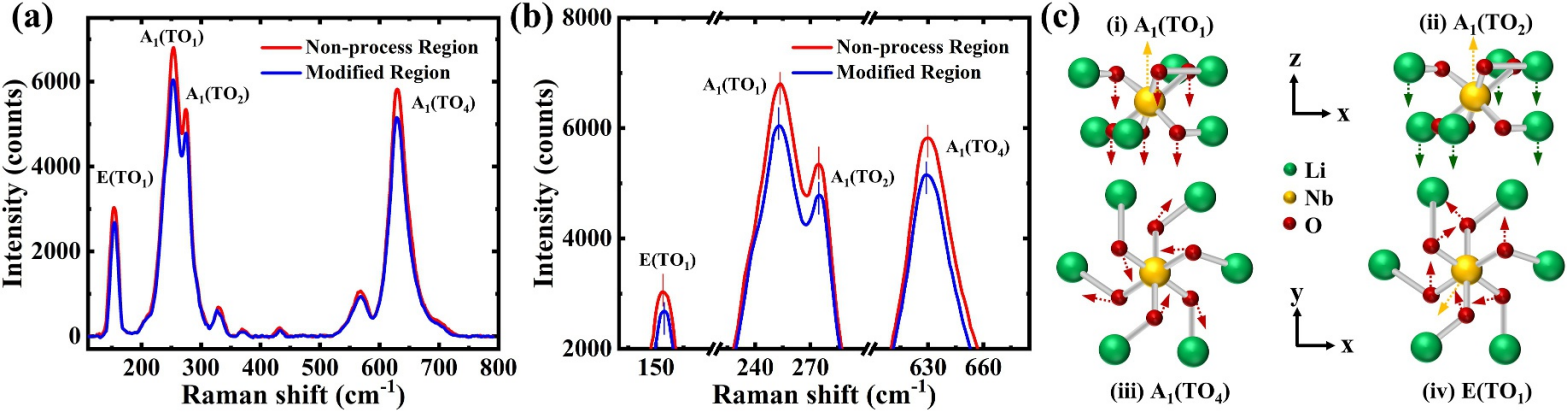
(a) Raman spectra of nonprocessed substrate and modification regions; (b) an enlarged view of the Raman spectra from the modification regions in panel (a); and (c) the schematic diagram of the phonon displacement patterns associated with Raman optical modes: (i) A_1_(TO_1_) mode, (ii) A_1_(TO_2_) mode, (iii) A_1_(TO_4_) mode, and (iv) E(TO_1_) mode.

To gain deeper insight into the lattice dynamics, Figure 2b shows an enlarged view of the Raman spectra from the modification regions in Figure 2a and the phonon displacement patterns corresponding to key Raman modes are schematically illustrated in Figure 2c. The mode at 252 cm^−1^ (A_1_(TO_1_)) is attributed to vibrations of Nb ions against oxygen octahedra along the *z*‐axis. After irradiation, this mode shows a slight red shift, implying a reduced interionic spacing within the [NbO_6_] units along the optical axis. In contrast, the A_1_(TO_2_) mode at 275 cm^−1^, associated with out‐of‐phase vibrations of Nb and Li ions parallel to the *z*‐axis, remains largely unchanged, indicating that the Nb‐Li distance is preserved. In the perpendicular plane, the A_1_(TO_4_) mode at 628 cm^−1^, related to in‐plane deformation of oxygen octahedra, exhibits a red shift within the modified track, suggesting compression of the octahedral structure. Meanwhile, the E(TO_1_) mode at 154 cm^−1^, involving Nb and Li vibrations confined to the x–y plane, displays a blue shift in the central irradiated zone, indicating increased interionic distance within the [NbO_6_] octahedra [31]. This response confirms that femtosecond laser modification induces highly localized and direction‐dependent lattice distortions, ultimately leading to the observed refractive index changes.

For the SHG measurements on the periodically modified regions, we performed SHG characterization under both normal incidence and oblique incidence. For the normal‐incidence measurements, a 1030 nm laser beam was focused using a 10X microscope objective (NA = 0.25, Olympus Inc.) and directed perpendicularly onto the LiNbO_3_ thin film along its *z*‐axis. The sample was intentionally defocused to broaden the irradiated area and ensure uniform exposure. Due to the weak SHG signal under normal incidence, which fell below the reliable detection limit of our spectrometer, the more sensitive CCD camera (33UX273, The Imaging Source Inc.) was employed to visualize and compare the spatial distribution of the second‐harmonic emission across different structures. A plano‐convex lens (*f* = 150 mm) was placed in front of the CCD to form an imaging system together with the objective. The second‐harmonic intensity distributions for normal incidence are presented as Supporting Information S1: Figure S2.

To further investigate the underlying mechanism of enhanced SHG in the laser‐modified structures, FDTD simulations were conducted under conditions corresponding to normal‐incidence SHG experiment as presented in Figure 3. The simulations were performed on a multilayer structure consisting of a 500 nm LiNbO_3_ film, a 2 μm SiO_2_ buffer layer, and a Si substrate. Simulations were carried out for both a pristine LiNbO_3_ surface and modified surfaces with systematically varied periods and diameters. A 1030 nm plane wave source was used under normal incidence on the LiNbO_3_ film, which was assigned a refractive index of *n* = 2.3243 at 1030 nm. The modified regions were assigned an increased refractive index of *n*_*mod*_ = 2.35, corresponding to Δ*n* ≈ 0.0257, which is consistent with previous studies that refractive index changes on the order of 10^−2^ can be achieved in LiNbO_3_ under certain irradiation conditions [32]. The evanescent field penetration depth at the fundamental wavelength can be estimated as $d\approx \lambda /\left(4\pi \sqrt{n_{\text{eff}}^{2}-1}\right)$, where *λ* = 1030 nm and the effective refractive index *n*_*eff*_ of the guided mode is approximately 2.2. This yields a penetration depth on the order of 41 nm. To ensure that the simulated modified region fully encompasses the evanescent field, we conservatively set the modification depth to 100–170 nm in the FDTD simulations, well above this estimated range. Interference fringes are observable in the simulated electric field distributions, resulting from the interaction between the incident light and the beam reflected from the silicon substrate. As shown in Figure 3b–f, enhanced optical field confinement is evident across all modified structures, attributable to a lens‐like focusing effect induced by the index‐modified zones. Among these, the structure with a period of 0.5 μm and a diameter of 0.5 μm, shown as Figure 3b, exhibits the strongest field enhancement. The enhanced focusing performance of the 0.5 μm‐period and 0.5 μm‐diameter structure is attributed to its optimal geometric parameters. A smaller diameter provides a sharper refractive index transition, effectively increasing the numerical aperture of the phase‐shifting zone and leading to tighter focal confinement. In contrast, larger diameters result in a more gradual phase gradient and reduced focusing efficiency. Simultaneously, the subwavelength period of 0.5 μm promotes stronger interaction and constructive interference with the incident 1030 nm wave, whereas larger periods exhibit reduced wave‐vector matching [33]. The combination of small period and small diameter therefore offers superior light concentration capability, leading to maximal local field enhancement and consequently the highest SHG intensity.

**FIGURE 3:**
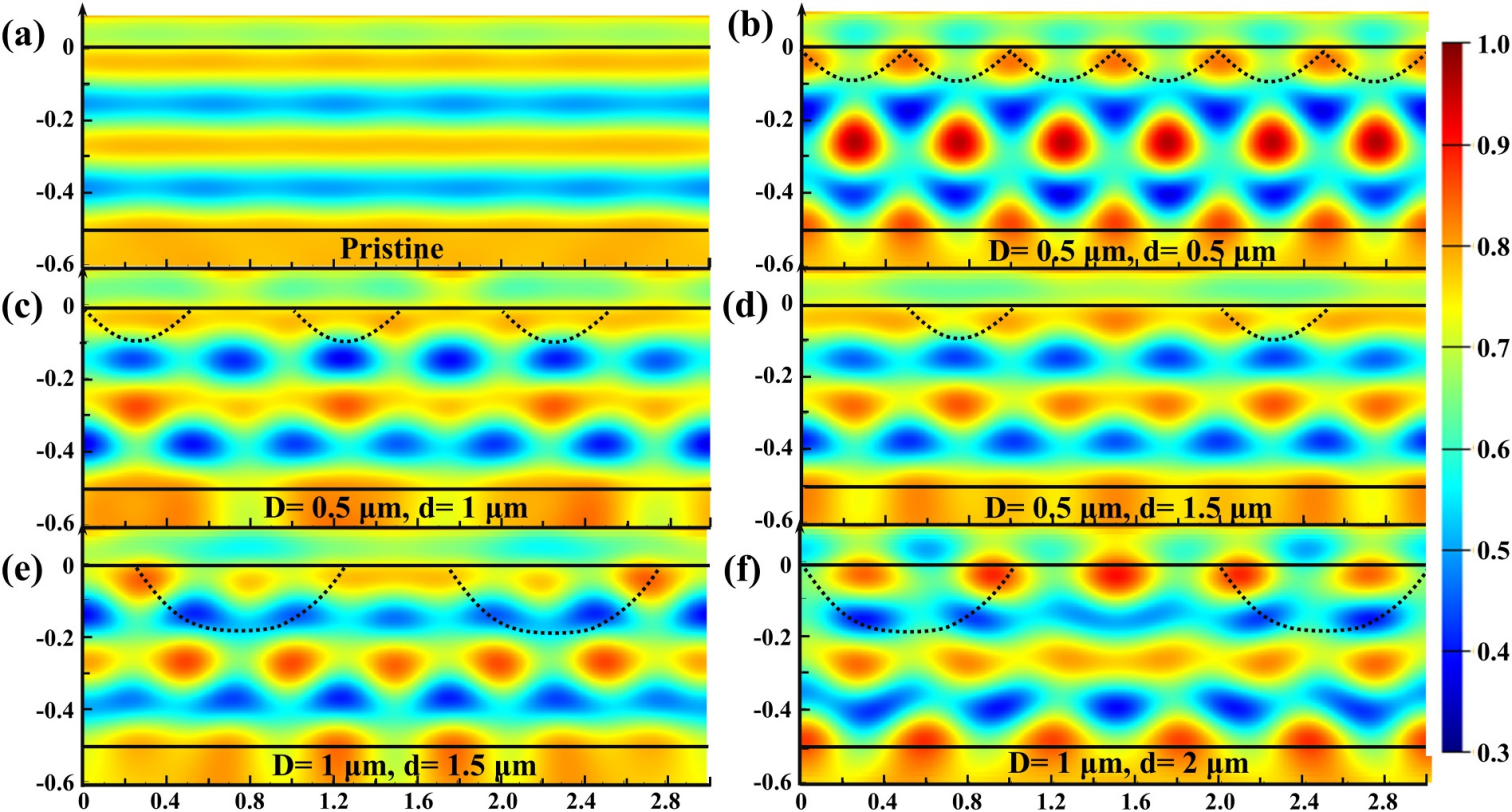
FDTD simulation of 1030 nm laser irradiation on (a) a pristine LiNbO_3_ surface; a modified LiNbO_3_ surface with (b) 0.5 μm period and 0.5 μm diameter, (c) 1 μm period and 0.5 μm diameter, (d) 1.5 μm period and 0.5 μm diameter, (e) 1.5 μm period and 1 μm diameter, and (f) 2 μm period and 1 μm diameter. The dashed line represents the structural cross‐section.

In addition to the normal‐incidence SHG measurements, the second‐harmonic intensity is also characterized under oblique incidence using a 1030 nm laser as shown in Figure 4a. Due to stronger SHG signal and easier collection under oblique excitation, the output is gathered via a spectrometer. The detailed test system is described in the Supporting Information S1. In the normal‐incidence SHG measurements, structures with a period of 0.5 μm/diameter of 0.5 and 1.0 μm/diameter of 0.5 μm were identified to exhibit strong focusing effects. Under oblique incidence, two complementary mechanisms contribute to the enhanced SHG. Firstly, the periodic arrangement of modified regions forms an effective index grating that satisfies the grating coupling condition, efficiently coupling the incident light into the guided modes of the LiNbO_3_ film [34]. Simultaneously, the subwavelength modified dots (diameter = 0.5 μm) may further enhance local fields through scattering effects [35]. The optimal combination of these effects was achieved with a period of 0.5 μm and dot diameter of 0.5 μm. Meanwhile, the angular dependence of the guided‐mode resonance was further investigated via FDTD simulations (Supporting Information S1: Figure S4), which show that the resonance is optimally excited at 45° for the pump wavelength of 1030 nm. Under oblique incidence at 45°, the SHG intensity of the optimal structure exhibits a 4.75‐fold enhancement compared to the unmodified film as shown in Figure 4b–c. Due to the polarization‐sensitive nature of the metasurface and the significant difference in χ^(2)^ tensor components along the *z*‐ and *y*‐crystal directions, the SHG intensity exhibits strong dependence on the pump polarization as shown in Figure 4d.

**FIGURE 4:**
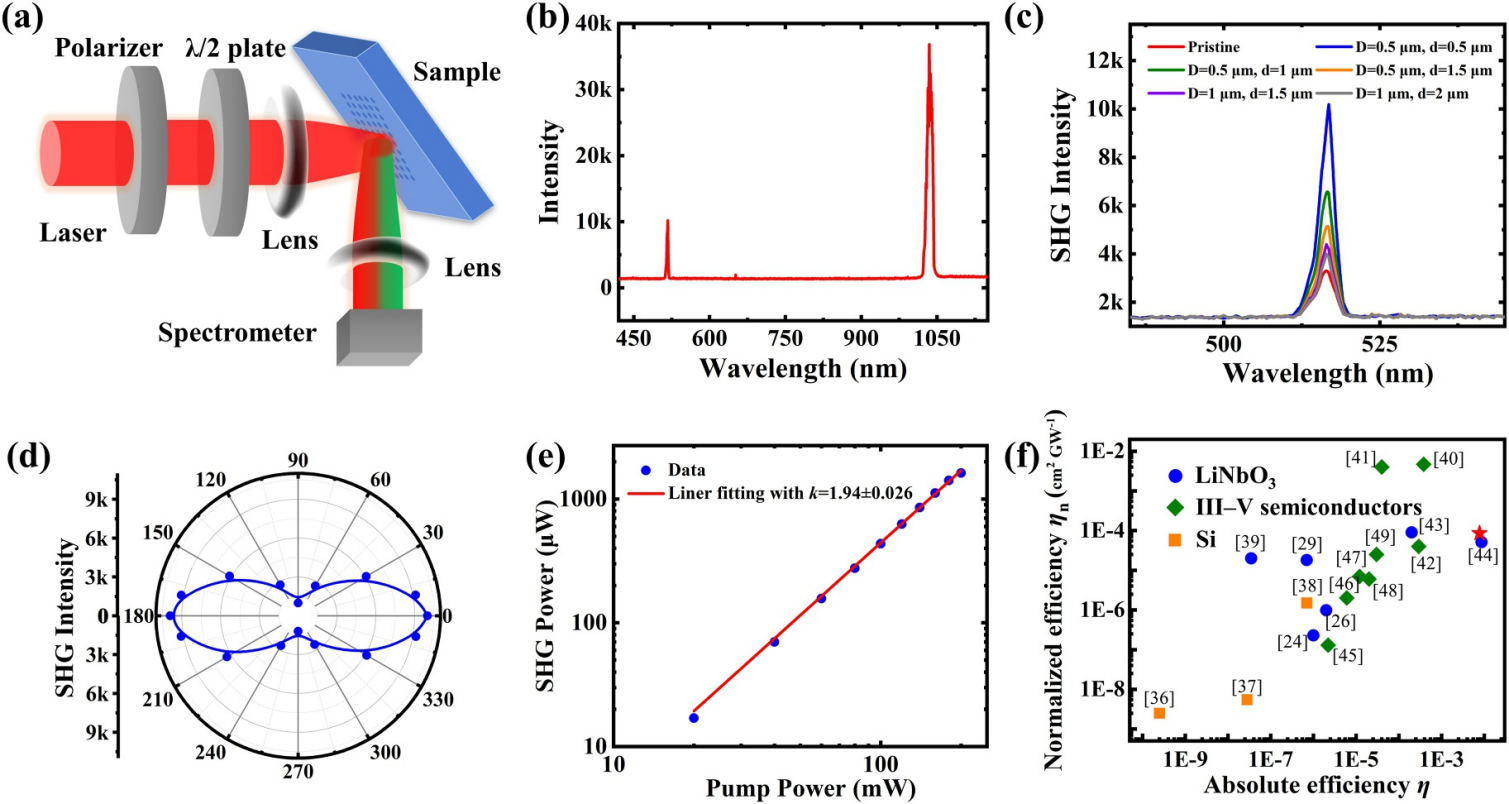
(a) Schematic of the SHG measurement; (b) normalized spectra of fundamental pumping laser at 1030 nm (right) and SHG signal at 515 nm (left) generated from the metasurface with 0.5 μm period and 0.5 μm diameter; (c) the measured SHG spectrum of the LiNbO_3_ modified surface with different structures and an unpatterned film, respectively; (d) measured SHG intensity as a function of the polarization angle of the pump laser beam; (e) SHG output power as a function of the incident pump power for the optimal structure under oblique incidence; and (f) comparison of SHG efficiency in Si, III–V semiconductors, and LiNbO_3_ surface [24, 26, 29, 36, 37, 38, 39, 40, 41, 42, 43, 44, 45, 46, 47, 48, 49].

Compared to normal‐incidence testing, the oblique excitation scheme combines longitudinal focusing enhancement from the modified regions with guided‐mode resonance effects in the transverse direction as illustrated in Supporting Information S1: Figure S3. This hybrid mechanism significantly amplifies the fundamental field and improves harmonic output directionality. Through quantitative power measurement of both incident and output beams, we evaluated the absolute conversion efficiency. A calibrated power meter (PM100D with S140C, Thorlabs) was used to measure the SHG power directly. Figure 4e presents the SHG output power as a function of the incident fundamental pump power. The SHG power exhibits a monotonic increase with increasing pump power across the measured range. The SHG power follows a quadratic dependence on the pump power as expected for a second‐order nonlinear process: $P_{\text{SHG}}\propto P_{\text{pump}}^{2}$. A linear fit to the log‐log plot yields a slope of approximately 1.94, confirming the quadratic nature of the SHG process. The average power of the fundamental beam at 1030 nm incident on the sample was determined to be *P*_FH_ = 180 mW. The fundamental beam at 1030 nm was blocked using a short‐pass filter (MVF52A, Thorlabs) and the SHG power was measured to be *P*_SH_ = 1.41 mW. The corresponding conversion efficiency was *η* = P_SH_/*P*_FH_ = 7.8 × 10^−3^. With a spot diameter of 500 μm measured by the knife‐edge method, the peak pump intensity *I*_*peak*_ was determined to be 0.09 TW cm^−2^. Since SHG efficiency scales with peak intensity, we used the normalized efficiency *η*_*norm*_ = *η*/*I*_*peak*_ = 8.7 × 10^−5^ cm^2^ GW^−1^ as a benchmark for device performance. As summarized in Figure 4f, this value exceeds those of many dielectric metasurfaces and is comparable to some high‐performance III–V semiconductor platforms, attributable to the large intrinsic χ^(2)^ of LiNbO_3_ combined with the high quality factor of the guided‐mode resonance achieved through precision femtosecond laser modification.

To further elucidate the enhancement mechanism under the oblique incidence configuration used in our main SHG experiments, we performed additional FDTD simulations at an incident angle of 45° as shown in Figure 5. Unlike the normal‐incidence case where enhancement arises primarily from a focusing effect, under oblique incidence the periodic structure can couple the incident light to guided modes of the LiNbO_3_ film. To accurately model the experimental conditions, the simulations included the 2 μm‐thick SiO_2_ buffer layer between the LiNbO_3_ film and the Si substrate, which isolates the waveguide from the high‐index substrate and enables the formation of high‐quality resonances.

**FIGURE 5:**
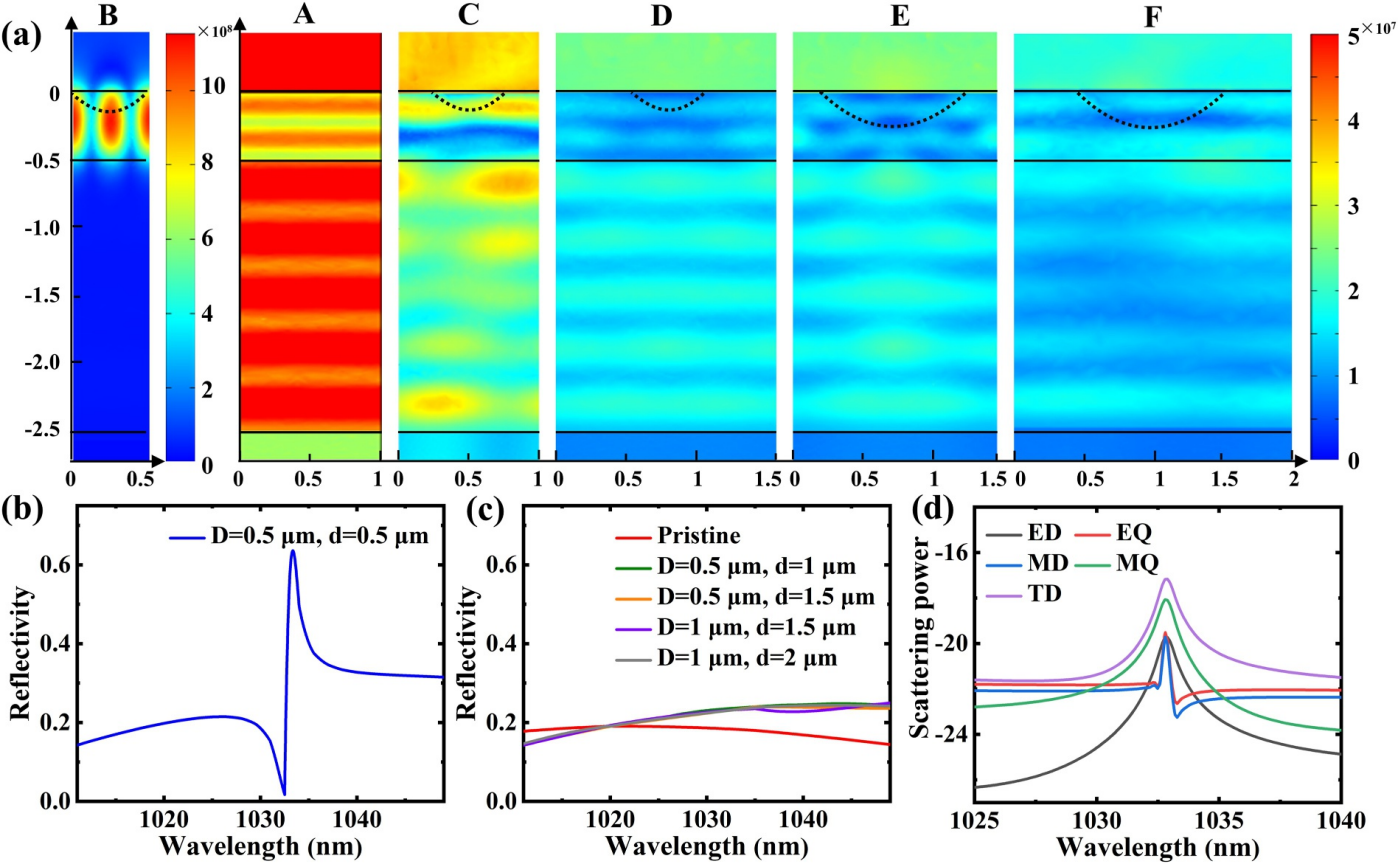
FDTD simulations of the metasurface under oblique incidence (*θ* = 45°). (a) Electric field intensity distributions at 1030 nm for the pristine LiNbO_3_ film A and five metasurface structures with different geometric parameters: 0.5 μm period and 0.5 μm diameter (B), 1 μm period and 0.5 μm diameter (C), 1.5 μm period and 0.5 μm diameter (D), 1.5 μm period and 1 μm diameter (E), and 2 μm period and 1 μm diameter (F); (b) simulated reflectivity spectrum of structure B as a function of wavelength; (c) reflectivity spectra of structures A, C, D, E, and F; and (d) multipole scattering analysis of structure B at the resonance wavelength (1033 nm). The scattering powers of different multipole moments are shown in arbitrary units.

As shown in Figure 5a, the electric field distribution at 1030 nm varies significantly with the geometric parameters of the metasurface. Consistent with the trend observed in the normal‐incidence FDTD results, structure B (period = 0.5 μm and diameter = 0.5 μm) exhibits the strongest field confinement, indicating optimal resonant coupling under oblique incidence. It is important to note that the strong electric field in the pristine film (A) arises from Fabry–Perot interference in the multilayer structure, not from guided‐mode resonances. Although this interference enhances the local field, it does not lead to strong SHG enhancement because the field distribution is not optimized for coupling to the χ^(2)^ nonlinearity. Figure 5b,c show the corresponding reflectivity spectra. A sharp Fano‐type resonance peak is observed exclusively for structure B, confirming the excitation of a guided‐mode resonance. It is noted that the simulated resonance peak is centered at approximately 1033 nm, which shows a slight offset of 3 nm from the pump wavelength of 1030 nm used in our experiments. This minor deviation is well within the typical tolerances expected from nanofabrication variations, including slight discrepancies in the actual structural dimensions (period and diameter) and the precise refractive index contrast of the laser‐modified regions. Importantly, the resonance linewidth is sufficiently broad to ensure significant field enhancement at 1030 nm. Furthermore, the broadband nature of our femtosecond laser pulse (spectral width 10 nm) efficiently excites the resonance, ensuring that the local field confinement at the pump wavelength remains strong. This resonance is absent in the other geometries (Figure 5c), demonstrating the critical role of structural parameters in achieving resonant enhancement.

To understand the physical origin of this resonance, we performed a multipole decomposition analysis at the resonance wavelength for structure B based on the multipole scattering theory [50] as shown in Figure 5d. The analysis reveals that the toroidal dipole (TD) moment is the dominant contributor to the scattering response, with its amplitude significantly outweighing the electric dipole (ED), magnetic dipole (MD), electric quadrupole (EQ), and magnetic quadrupole (MQ) contributions. The strong toroidal dipole response, known to produce sharp Fano resonances with high quality factors, is therefore identified as the primary mechanism behind the guided‐mode resonance observed in our optimal structure. This resonant field enhancement under oblique incidence, combined with the focusing effect under normal incidence demonstrated in Figure 3, provides a comprehensive explanation for the 4.75‐fold enhancement in SHG efficiency observed experimentally.

## 4 Conclusion

In conclusion, we have demonstrated a one‐step nonablative femtosecond laser processing technique for direct writing of periodic subwavelength structures on LiNbO_3_ thin films. This method effectively minimizes surface damage and suppresses microcrack formation, outperforming conventional ablation‐based approaches and preserving the material's intrinsic optical quality. The designed periodic structures were shown to facilitate efficient second‐harmonic generation through two complementary mechanisms. Under normal incidence, the laser‐modified regions induce a longitudinal focusing effect that concentrates the optical field, contributing to enhanced nonlinear conversion. Under oblique incidence, the periodic index modulation enables grating coupling to guided‐mode resonances, resulting in strong in‐plane field confinement. The optimal combination of these effects yields a 4.75‐fold enhancement in SHG efficiency under 1030 nm femtosecond laser irradiation at oblique incidence. The underlying mechanisms—including the refractive index modification, resonant behavior and field enhancement—were systematically investigated through FDTD simulations and Raman spectroscopy, providing insight into the light–matter interactions and structural changes induced by femtosecond laser modification. This work establishes a straightforward low‐damage fabrication strategy for high‐efficiency nonlinear photonic devices. The proposed technique holds great promise for applications in integrated photonic circuits, on‐chip wavelength conversion, and scalable metasurface‐based light sources.

## Author Contributions

**Feifei Wang:** methodology, writing – review and editing, project administration, writing – original draft, funding acquisition. **Yuntao Lang:** investigation, writing – original draft, formal analysis, methodology, data curation. **Changji Pan:** writing – review and editing, methodology, validation, formal analysis. **Yang Lu:** writing – review and editing, validation, supervision. **Xiaozhe Chen:** validation, writing – review and editing, supervision. **Zhe Peng:** validation, writing – review and editing, methodology, formal analysis. **Liandong Yu:** conceptualization, funding acquisition, methodology, writing – review and editing, project administration.

## Funding

This study was funded by the National Key R&D Program of China (Grant 2023YFB4605101), National Natural Science Foundation of China (Grants 52305595, U22A20205, and 52575647), Natural Science Foundation of Shandong Province (Grant ZR2023QE169), and the Fundamental Research Funds for the Central Universities (Grant 22CX06062A).

## Conflicts of Interest

The authors declare no conflicts of interest.

## Supporting information


Supporting Information S1

## Data Availability

Data underlying the results presented in this paper are not publicly available at this time but may be obtained from the authors upon reasonable request.
