# Supplementary material for: One‐Step Fabrication of Non‐Ablative Microstructures for Efficient Second Harmonic Generation on LiNbO3 Thin Film
Source: Nanophotonics. 2026 Apr 30;15(9):e70096. doi: 10.1002/nap2.70096 (PMC13642112; doi:10.1002/nap2.70096)
Supplement: Supplementary file 1 [file NAP2-15-e70096-s001.docx]

Supporting Information

One-step fabrication of non-ablative microstructures for efficient second harmonic generation on LiNbO_3_ thin film

Feifei Wang, Yuntao Lang, Changji Pan,* Yang Lu, Xiaozhe Chen, Rongke Gao, Zhe Peng, and Liandong Yu*

F. F. Wang 1, Y. T. Lang 1, Y. Lu 1, X. Z. Chen 1, R. K. Gao 1, Z. Peng 1, L. D. Yu 1

College of Control Science and Engineering, China University of Petroleum (East China), Qingdao, China
E-mail: liandongyu@upc.edu.cn

C. J. Pan 2, 3
School of Mechanical Engineering and Automation, Fuzhou University, Fuzhou, China

Department of Physics, ETH Zurich, Zurich, Switzerland

E-mail: chapan@phys.ethz.ch

**1. Metasurface morphology**

Metasurfaces with five periods are fabricated by femtosecond laser modification, as shown in **Figure S1**. The period is d and the diameter is D.

**Figure S1.** Metasurfaces with five periods fabricated by femtosecond laser modification

**2. SHG measurements for normal incidence**

**Figure S2** presents the second-harmonic intensity distributions for both unmodified and periodically modified regions on the LiNbO_3_ film after femtosecond laser processing. The x-axis corresponds to the gray level intensity, and the y-axis represents the pixel count at each gray level. The inset image in the figure is the second harmonic image for both unmodified and periodically modified regions captured by CCD. Figure S2(a) displays the reflected SHG signal from an unprocessed area, where a detectable second-harmonic signal is observed due to the intrinsic nonlinearity of LiNbO_3_, with most pixel values clustering around 200. In contrast, Figure S2(b-f) correspond to structures with periods ranging from 0.5 μm to 2 μm and feature diameters between 0.5 μm and 1.5 μm. These regions exhibit a distinct shift in gray level distribution toward higher values, around 225, indicating enhanced SHG emission. Key statistics including the average gray level and pixel proportion were extracted from each image and summarized in **Table S1**. The average gray values of the structured regions (B–F) are consistently higher than that of the unmodified area (A), confirming that the laser-induced periodic structures could enhance second-harmonic generation.

**Figure S2.** Schematic representation of the extracted SHG intensities (a) Pristine LiNbO_3_ film; Modified LiNbO_3_ film with (b) 0.5 μm period and 0.5 μm diameter, (c) 1 μm period and 0.5 μm diameter, (d) 1.5 μm period and 0.5 μm diameter, (e) 1.5 μm period and 1 μm diameter, (f) 2 μm period and 1 μm diameter. The inset image in the figure is the second harmonic image captured by CCD.

**Table S1.** Statistical analysis of grayscale value distribution extracted from Figure 2s

| Parameter | A | B | C | D | E | F |
| --- | --- | --- | --- | --- | --- | --- |
| Average gray level | 113.9 | 128.9 | 126.1 | 123.0 | 121.1 | 121.5 |
| Total brightness of pixels with gray level | 824,974,4 | 128,655,01 | 117,959,18 | 107,985,95 | 993,175,2 | 100,658,89 |

**3. Explanation of the enhancement mechanism under oblique incidence**

Compared to normal-incidence testing, the oblique excitation scheme combines longitudinal focusing enhancement from the modified regions with guided-mode resonance effects in the transverse direction, as illustrated in **Figure S3**. Under normal incidence, the laser-modified regions act as microlens-like structures due to the local increase in refractive index. This focusing effect persists under oblique incidence, concentrating the optical field along the propagation direction. Although this effect alone is relatively weak, it contributes to the overall field enhancement. Meanwhile, the periodic arrangement of modified regions forms an effective index grating in the transverse plane under oblique incidence. The incident light, which now has an in-plane wavevector component, can be coupled into the waveguide modes of the LiNbO_3_ film when the grating period matches the effective wavelength of the guided mode. For our optimally designed structure (period = 0.5 μm, diameter = 0.5 μm), this coupling condition is satisfied at an incident angle of 45°. The coupled light propagates laterally within the film, forming a guided-mode resonance that strongly confines the optical field in the transverse plane.

The oblique incidence configuration enables the simultaneous activation of both mechanisms: the longitudinal focusing effect concentrates the field along the propagation direction, while the guided-mode resonance provides strong transverse confinement. The resulting three-dimensional field localization significantly amplifies the local electric field, which in turn enhances the SHG efficiency by a factor of 4.75 compared to the unmodified film.

**Figure S3.** Principle of SHG with focusing and guided-mode resonance

**4. Method of** **SHG efficiency measurement**

For the nonlinear harmonic generation measurement, the excitation laser beam was with a 1030 nm wavelength, a 190-fs pulse duration, a 10 kHz repetition rate. A linear polarizer and a half-wave plate were used to adjust the polarization of the pump laser. The laser was focused onto the sample with a spot radius of 30 μm, using a lens with a focal length of 150 mm. The generated second-harmonic signals were collected by a lens with a focal length of 150 mm. The harmonic signal was finally coupled into a UV-IR grating spectrometer (AUT-EQ2000, Aunion Tech Ltd.) for spectrum analysis.

To avoid laser-induced damage to the LiNbO_3_ thin film while maintaining sufficient SHG signal, the focusing condition was slightly defocused. The beam was focused using a lens with a focal length of 150 mm, and the sample was positioned slightly away from the focal plane to achieve a spot radius of approximately 250 μm (measured by the knife-edge method). Under this condition, with an average power of 180 mW, the calculated peak intensity is 0.09 TW/cm², which is well below the damage threshold of the material.

**5. FDTD simulations of the reflectivity spectra across incident angles**

For angles below 45°, the resonance peak is not observed within the simulated wavelength range (950–1100 nm). The guided-mode resonance condition requires sufficient in-plane wavevector matching, which is not satisfied at smaller incident angles for our structure design. As the incident angle increases from 45° to 65°, the resonance peak progressively shifts from approximately 1033 nm toward shorter wavelengths, reaching around 980 nm at 65°. This angular dispersion is characteristic of guided-mode resonances and confirms the nature of the observed resonance. The simulations demonstrate that the resonance condition is optimally satisfied at 45° for our pump wavelength of 1030 nm.

**Figure S4.** FDTD simulations of the reflectivity spectra for the optimal structure across incident angles ranging from 45° to 65°
